# Impact of trichiasis surgery on daily living: A longitudinal study in Ethiopia

**DOI:** 10.12688/wellcomeopenres.11891.2

**Published:** 2017-12-06

**Authors:** Esmael Habtamu, Tariku Wondie, Sintayehu Aweke, Zerihun Tadesse, Mulat Zerihun, Berhanu Melak, Bizuayehu Gashaw, Kelly Callahan, Paul M. Emerson, Robin L. Bailey, David C.W. Mabey, Saul N. Rajak, Hannah Kuper, Sarah Polack, David Macleod, Helen A. Weiss, Matthew J. Burton

**Affiliations:** 1 The Carter Center, Addis Ababa, Ethiopia; 2 International Centre for Eye Health, London School of Hygiene & Tropical Medicine, London, WC1E 7HT, UK; 3 Amhara Regional Health Bureau, Bahirdar, Ethiopia; 4 The Carter Center, Atlanta, GA, 30306, USA; 5 International Trachoma Initiative, Atlanta, GA, 30030, USA; 6 Clinical Research Department, London School of Hygiene & Tropical Medicine, London, WC1E 7HT, UK; 7 International Centre for Evidence in Disability, London School of Hygiene & Tropical Medicine, London, WC1E 7HT, UK; 8 MRC Tropical Epidemiology Group, London School of Hygiene & Tropical Medicine, London, WC1E 7HT, UK

**Keywords:** Trachoma, Trachomatous Trichiasis, Surgery, Impact, Activity, Participation, Difficulty, Productivity

## Abstract

**Background:** Trachomatous trichiasis (TT) may lead to disability, impeding productive activities, resulting in loss of income. This study was conducted to determine if trichiasis surgery improves participation in productive and leisure activities, and ability to perform activities without difficulty or assistance.

**Methods:** We recruited 1000 adults with trichiasis (cases) and 200 comparison participants, matched to every fifth trichiasis case on age (+/- two years), sex and location. The ‘Stylised Activity List’ tool, developed for the World Bank Living Standard Measurement Survey, was adapted to collect data on activity in the last week (participation in activity, difficulty with activity, requirement of assistance for activity), at baseline and 12 months later. All trichiasis cases received trichiasis surgery at baseline. Random effect logistic regression was used to compare cases and comparison participants.

**Results: **There was strong evidence that trichiasis surgery substantially improves the ability of trichiasis cases to perform all the productive and leisure activities investigated without difficulty, with large increases in processing agricultural products, 21.1% to 87.0% (p<0.0001), farming, 19.1% to 82.4% (p<0.0001), and fetching wood, 25.3% to 86.0% (p<0.0001). Similarly, there was a significant increase in the proportion of cases who could perform activities without assistance, with the largest increases in animal rearing 54.2% to 92.0% (p<0.0001) and farming 73.2% to 96.4% (p<0.0001). There was no change in the proportion of comparison participants performing activities without difficulty or assistance. The change in most of the activities in cases was independent of visual acuity improvement and recurrent TT at 12 months. One year after trichiasis surgery, the proportion of cases reporting ocular pain reduced from 98.9% to 33.7% (p<0.0001).

**Conclusions: **Eyelid surgery for TT improves functional capabilities regardless of vision gains. These data lend strong support to the view that TT surgery improves function and contributes to improved household income and wealth.

## Introduction

Trachomatous trichiasis (TT) is the in-turning of the eyelashes towards the eye, which results from progressive conjunctival scarring caused by recurrent infection with *Chlamydia trachomatis.* Trichiasis causes painful abrasion of the cornea, leading to corneal opacification and usually irreversible visual impairment. Approximately 3.2 million people have un-treated trichiasis, and 2.4 million people are visually impaired from trachoma worldwide, of whom 1.2 million are estimated to be irreversibly blind, making trachoma the leading infectious cause of blindness worldwide ^1– 3^.

The pain and photophobia from trichiasis may also lead to disability, limiting engagement with productive household and agricultural activities even prior to the development of visual impairment. This can result in loss of income and additional economic pressure on often already deprived households. We have previously reported that TT significantly reduces participation in productive household, outdoor, agricultural and leisure activities, even prior to the development of visual impairment ^4^. Moreover, we found that TT cases experienced considerably more difficulty in performing tasks and required extra assistance, compared to their neighbours without TT ^4^. Other studies have shown that trichiasis causes considerable functional and physical impairment, inability to work and earn an income ^5– 7^.

The economic impact of TT affects not just the individual but potentially the whole family. In most trachoma endemic settings, employment opportunities are often limited and household income is mainly generated from agricultural activities. These are carried out by all family members regardless of age and gender. For instance women, who are more frequently affected by TT than men, usually participate in both household and agricultural activities, including caring for family members, cooking, farming and processing agricultural products ^8^. In Sub-Saharan Africa, agriculture accounts for nearly 60% of employment of women ^9^.

TT is usually treated with corrective eyelid surgery to stop the abrasive damage to the cornea with the aim of reducing the risk of sight loss ^10^. However, the surgery also improves overall wellbeing and the individual’s capacity to engage in household and agricultural activities by effectively treating the pain and discomfort from trichiasis ^11^. A longitudinal study in Ethiopia assessed the six-month effect of trichiasis surgery on physical functioning using a locally appropriate questionnaire. This study found that trichiasis surgery increased the proportion of trichiasis patients performing physical activities without difficulty ^7^. Other than this, there are no longitudinal studies measuring the long-term effect TT surgery has on engagement and execution of activities.

The ‘Stylised Activity List’ was developed for the World Bank’s Living Standards Measurement Survey (LSMS) to assess participation in various productive and leisure activities before and after interventions ^12^. We have previously reported a case-control study, which adapted this tool to compare a subset of TT cases to controls without TT ^4^. The current additional longitudinal comparative study was undertaken to explore the long-term impact of trichiasis surgery on productive and leisure activity participation, difficulty and required assistance in TT cases, and compare this with the same controls used in the baseline paper (hereafter referred to as comparison participants).

## Methods

### Ethical statement

This study was reviewed and approved by the National Health Research Ethics Review Committee of the Ethiopian Ministry of Science and Technology (Reference number, 3.10/573/06), the London School of Hygiene & Tropical Medicine (LSHTM) Ethics Committee (Reference number, 6424), and Emory University Institutional Review Board (Reference number, IRB00067868). Written informed consent in Amharic was obtained prior to enrolment from participants. It was conducted in accordance with the Declaration of Helsinki. If the participant was unable to read and write, the information sheet and consent form were read to them and their consent recorded by thumbprint. Interviews were conducted privately, paper data were archived in a locked cabinet and electronic data were stored on a password-protected computer isolated from the Internet in a secure dedicated study office. Study participants with identified ocular problems were managed as per local protocol. 

### Study design and participants

This longitudinal study was nested within a clinical trial of two alternative surgical treatments for TT ^13^. The study design and participants in this study has been described previously ^4, 14^. In summary, we recruited 1000 TT cases into the trial, who were also enrolled into this impact study. Cases were defined as individuals with one or more eyelashes touching the eyeball or with evidence of epilation in either or both eyes in association with tarsal conjunctival scarring. They were identified mainly through community-based screening ^15^. Recruitment was done in three districts of West Gojam Zone, Amhara Region, Ethiopia between February and May 2014.

We also recruited 200 comparison participants. These were individuals without clinical evidence or a history of trichiasis (including epilation), who came from households without a family member with trichiasis or a history of trichiasis. Comparison participants were individually matched to every fifth trichiasis case by location, sex and age (+/- two years). The research team visited the sub-village (30–50 households) of the trichiasis case that required a matched control. A list of all potentially eligible people living in the sub-village of the case was compiled with the help of the sub-village administrator. One person was randomly selected from this list using a lottery method, given details of the study and invited to participate if eligible. If a selected individual refused or was ineligible, another was randomly selected from the list. When eligible comparison participants were not identified within the sub-village of the index case, recruitment was done in the nearest neighbouring sub-village, using the same procedures.

### Baseline assessment

Data from TT cases were collected at health facilities at the time of enrolment into the clinical trial, prior to trichiasis surgery. Data from the comparison participants were collected at their homes. Six trained Amharic speaking interviewers collected data from participants using a standardised questionnaire, including socio-demographic variables (age, sex, marital and literacy status), presence of any other health problems in the last month and self-rated socioeconomic status (SES). For self-rated SES, participants were asked to rate the wealth of their household in relation to other households in their village by choosing one of the following options: (1) very poor, (2) poor, (3) average, (4) wealthy or (5) very wealthy ^4^.

### Activity participation data

The 'Stylised Activity List' tool developed for the World Bank Living Standard Measurement Survey was used to collect activity participation data ^12^.

This tool contains a list of common activities in different subgroups: household activities, paid work, work for own use, leisure activities and personal activities. Participants were asked if they had participated in each of the activities in the subgroups in the last week. If they had undertaken a specific activity in the last week, they were asked the question *“How much difficulty did you have in doing [Activity] in the last week?”* and asked to choose one of the following options: (0) extreme/not able to do, (1) a lot of difficulty, (2) some difficulty, (3) little difficulty, (4) no difficulty; and another question whether they have done the activity: (1) fully assisted, (2) with some assistance, (3) with no assistance.

### Ocular pain impact data

Data on the impact of ocular pain on daily living were collected through a locally relevant structured questionnaire. This was developed through a focus group discussion with community based TT case screeners (Eye Ambassadors), and then was piloted in two surgical outreaches. Both cases and comparison participants were asked the question: “How often have you experienced eye pain in the last month?”, then they were asked to choose an option from a four point scale: “Never”, “Occasionally”, “Often”, “Constantly”. Those who reported experiencing any pain in the last month were asked the following five questions, using the same four point scale options (above): *“(1) how often has eye pain interfered with your personal care such as bathing, eating, and dressing?”; “(2) how often has eye pain disturbed your sleep?”; “(3) how often has eye pain interfered with your household work such as cooking, house cleaning, washing clothes, fetching water, fetching firewood, caring for other family members?”; “(4) how often has eye pain affected your agricultural or paid work?”, “(5) how often has eye pain affected your participation in social activities such as attending weddings, social meetings, and funerals?”*

### Clinical data

Presenting LogMAR (Logarithm of the Minimum Angle of Resolution) visual acuity at two metres was measured using “PeekAcuity” software on a Smartphone in a dark room for both cases and comparison participants ^16^. An ophthalmic examination was conducted using a 2.5x binocular magnifying loupe and a bright torch.

### Surgical intervention

Immediately after baseline data collection was completed, all cases received trichiasis surgical management. They were randomised to receive either the bilamellar or the posterior lamellar tarsal rotation, which were being compared in the clinical trial ^13^. Both surgical procedures involve an incision through the scarred upper eyelid, parallel to and about 3mm above the lid margin, followed by outward rotation and suturing in the corrected position ^17^. Six standardised trichiasis surgeons performed the surgery.

### Follow-up assessment

Follow-up was conducted approximately one year after enrolment (minimum 10 and maximum 14 months), during the same season as the baseline assessment. For cases, a short reminder letter was sent to attend the 12-month follow-up. Follow-up data were collected for the majority of cases at a health facility. For cases that could not come to the health facility, data were collected during a home visit. Follow-up data were collected on comparison participants at their homes. Participants were interviewed using the same ‘Stylised Activity List’ and ocular pain impact tool as at baseline, and clinical data were collected using the same procedures by the same interviewers and clinical grader.

### Analysis

Sample size determination has been reported previously ^11, 14^. Data were double entered into Access (Microsoft), cleaned in Epidata 3.1 and transferred to Stata 11 (StataCorp) for analysis. Analyses were restricted to participants with both baseline and follow-up data. 

The three binary primary outcomes assessed were “participated in activity in last week”, “performed activity without difficulty in last week” and “performed activity without any assistance in last week”. To generate the latter two outcomes, the “difficulty” and “assistance” question responses were categorised as follows. The “difficulty” question responses were dichotomized into “performed activity with difficulty” (if the participant was not able to do it, had a lot of difficulty, or some difficulty in doing the activity in the last week); and “performed activity without any difficulty” (if the participant had no difficulty in doing the activity in the last week). The “assistance” question responses were dichotomized as “performed activity with assistance” (if the participant performed activity with some assistance or was fully assisted in the last week) and “performed activity without any assistance” (if the participant performed the activity without any assistance).

For ease of presentation, activities were regrouped into productive household activities (cooking and cleaning dishes, house cleaning, washing clothing and looking after family members), productive outdoor activities (animal rearing, farming, processing agricultural products, fetching wood, fetching water, shopping/marketing, travelling), paid work (daily laboring and self employment activities), leisure/social activities (making social or family visits, attending ceremonies, attending social meetings, engagement in relaxing activities, such as reading, watching TV, listening to the radio or chatting with friends), and daily activities (eating, bathing, dressing and sleeping). For the combined analysis of these activities in their subgroups, participation was determined by whether an individual participated in at least one of the activities in the subgroup during the last week. Being without difficulty or not requiring assistance was determined at the subgroup level by whether an individual could perform at least one task within the subgroup without reported difficulty or without assistance.

The vision data analyses have been reported previously in detail ^11^. Participants were grouped into better vision (improvement of >0.1 LogMAR), same vision (-0.1 to 0.1 LogMAR) and worse vision (deterioration of >0.1 LogMAR) categories in relation to their baseline vision scores. Based on their baseline trichiasis severity, cases were categorized into Minor Trichiasis (<6 lashes or evidence of epilation in <1/3rd of the lashes) and Major Trichiasis (≥ 6 lashes or evidence of epilation in ≥ 1/3rd of the lashes).

To quantify the difference in the proportion of cases who participate in an activity at baseline and the proportion at follow up, a two-sample test of proportion was employed, providing an estimate of the differences in proportion with a 95% confidence interval. This was also performed separately for the comparison group. The same procedure was also applied to compare the proportions of participants with difficulty and requiring assistance at baseline and follow up, among those who report participation in the activity in the last week.

To test whether the increase/decrease in proportion of participants from baseline to follow up in the three outcome measures differed in the cases and comparison group, a random effect logistic regression model was used, with case/non-case status and time point (baseline or follow up) as exposure variables. This model was adjusted for the matching variables (age and sex), and presence of another health problem during the last month, as these factors may confound activity participation, difficulty and assistance requirements. The analysis was not adjusted for village, as neighbourhood comparison participants were used, and it was assumed that village would not affect participation, difficulty and assistance in activities.

An interaction was included between case status and time point, with the p-value for that interaction reported to provide the strength of evidence that the odds ratio relating the odds of participation, doing activity without difficulty and assistance between baseline and follow-up differed between cases and non-cases. These tests of interaction between case/comparison status and time point in the difficulty and assistance data were not possible to analyse in all activities, as some of the proportion changes (between baseline and follow-up) in the comparison participants have inadequate variability. Therefore, in such activities, data analysis was restricted to within the cases to show if any increases or decreases in proportion of cases doing an activity without difficulty and assistance differ between baseline and follow-up. In a further stratified analysis, p-values for interaction between visit and vision change at 12-month follow-up, and visit and TT recurrence status in cases alone were generated to show if any increase or decrease in proportion of cases performing an activity without difficulty and assistance show a trend across the three group of vision change classifications (better, same and worse); and to see the effect of recurrent TT on activity participation, difficulty and assistance. 

A similar two-sample test of proportion analysis was performed to examine whether there was a significant change in the proportion of cases and comparison participants with ocular pain, and its impact on personal care, sleep, household, paid, agricultural and social activities. For this analysis among the four response options in the ocular pain impact data; “occasionally”, “often” and “constantly” were combined to create a binary variable with the “Never” option. Random effect ordinal logistic regression model was used to show if there is a trend of increase or decrease in the proportion of cases and comparison participants (separately) with negative reported impact of ocular pain (ordered variable as none, occasionally, often and constantly) on daily living between baseline and follow-up. Then p-values for interaction between visit and case/comparison status were calculated to show if any increase or decrease in proportion of participants with no pain or no negative impact of ocular pain on daily living between baseline and follow-up significantly differ between cases and comparison participants. P-value <0.05 was considered statistically significant in this study.

## Results

### Demographic and clinical characteristics

At baseline, 1000 TT cases and 200 comparison participants were recruited. At the 12-month follow-up, complete activity participation data were collected from 980 (98%) cases and 198 (99%) comparison participants. The baseline demographic and clinical characteristics of cases and comparison participants seen at 12-months have been reported previously ^11^. In summary, cases and comparison participants were adequately matched for age, but there were significantly more females among the comparison participants (84.3%) than the cases (76.4%, p=0.02). Compared to the comparison participants, the trichiasis cases were more likely to be illiterate (p=0.008), widowed or divorced (p<0.0001), be from poorer households (p<0.0001), and report another health problem in the past month (p<0.0001). The majority of the comparison participants (97%) had normal vision (≥6/18), while about 36% of cases had visual impairment (<6/18) and significantly lower contrast sensitivity score (p<0.0001).

### Activity participation

Between baseline and one year after surgery there was a significant increase in the proportion of cases participating in activities during the previous week for five of the seven productive outdoor activities assessed: farming by 5.7% (95% CI, 1.3–10.3%; p=0.01), processing agricultural products by 18.1% (95% CI, 13.7–22.4%; p<0.0001), fetching wood by 23.6% (95% CI, 19.3–27.9%; p<0.0001), fetching water by 5.4% (95% CI, 1.6–9.3%; p=0.006) and traveling by 9.5% (95% CI, 5.1–13.8%, p<0.0001). The only other activity that showed a marked increase in participation among cases was the leisure/social activity attending ceremonies, which increased by 27.5% (95% CI, 23.4–31.6%; p<0.0001).

However, when the relative changes in activity participation among the cases and the comparison participants are compared, there are few marked differences ( Table 1). To analyse this, we tested the relative likelihood of cases and comparison participants who did not participate in a specific activity at baseline subsequently participating in the activity at 12-months. For most activities there was no difference (p values in Table 1). The exceptions were firstly fetching wood, which was slightly more frequent among the cases (borderline significance, p=0.06); secondly, processing agricultural products (p=0.02), looking after a family member (p=0.0002) and making social visits (p=0.0002) were relatively more frequent in the comparison participants at 12-months; thirdly participation in washing clothes reduced in both groups at 12-months and was slightly less frequent in the comparison participants (borderline significance, p=0.05) ( Table 1). 

**Table 1.** Change in activity participation of cases and comparison participants between baseline and 12-month follow-up.

Activity	Performed activity in the last week						
	*Baseline*		*Follow-up*		*Diff*	*(95% CI)*	*P-value ^a^*
	*n*	*(%)*	*n*	*(%)*			
***Productive household activities*** Cooking and cleaning dishes Cases CPs House cleaning Cases CPs Washing clothing Cases CPs Looking after family member Cases CPs	740 168 708 167 382 129 673 136	(75.5) (84.8) (72.2) (84.3) (39.0) (65.1) (68.7) (68.7)	714 165 707 165 373 109 706 170	(72.9) (83.3) (72.1) (83.3) (38.1) (55.0) (72.0) (85.9)	-2.6 -1.5 -0.1 -0.1 -0.9 -10.1 3.3 17.2	(-6.53 – 1.22) (-0.87 – 5.67) (-0.40 – 0.39) (-0.83 – 0.62) (-5.22 – 3.39) (-19.7, -0.51) (-0.67 – 7.41) (9.09 – 25.2)	0.95 - 0.05 0.0002
***Productive outdoor activities*** Animal rearing Cases CPs Farming Cases CPs Processing agricultural products Cases CPs Fetching wood Cases CPs Fetching water Cases CPs Shopping/marketing Cases CPs Travelling Cases CPs	675 163 466 118 455 159 376 152 703 170 563 151 365 116	(68.9) (82.3) (47.6) (59.6) (46.4) (80.3) (38.4) (76.8) (71.7) (85.9) (57.4) (76.3) (37.2) (58.6)	666 163 522 162 632 190 607 168 756 185 534 143 458 140	(68.0) (82.3) (53.3) (81.8) (64.5) (96.0) (61.9) (84.8) (77.1) (93.4) (54.5) (72.2) (46.7) (70.7)	-0.9 0.0 5.7 22.2 18.1 15.7 23.6 8.0 5.4 7.5 -2.9 -4.1 9.5 12.1	(-5.03 – 3.20) - (1.29 – 10.3) (13.5 – 30.9) (13.7 – 22.4) (9.47 – 21.8) (19.3 – 27.9) (3.64 – 15.8) (1.55 – 9.26) (1.62 – 13.5) (-7.53 – 1.43) (-12.6 – 4.56) (5.14 – 13.8) (2.78 – 21.5)	0.80 - 0.02 0.06 0.08 0.70 0.52
***Paid work*** Daily labouring Cases CPs Self employment ^†^ Cases CPs	47 4 147 25	(4.80) (2.02) (15.0) (12.6)	38 1 151 27	(3.88) (0.51) (15.4) (13.6)	-0.92 -1.5 0.4 1.0	(-2.72 – 0.88) (-3.71 – 0.68) (-2.77 – 3.59) (-5.64 – 7.66)	0.27 0.80
***Leisure/Social activities*** Social visits Cases CPs Attending ceremonies Cases CPs Attending social meetings Cases CPs Relaxing activities ^b^ Cases CPs	683 148 235 59 107 31 186 64	(69.7) (74.7) (24.0) (29.8) (10.9) (15.7) (19.0) (32.3)	702 181 504 103 131 30 210 63	(71.6) (91.4) (51.5) (52.0) (13.4) (15.2) (21.4) (31.8)	1.9 16.7 27.5 22.2 2.5 -0.5 2.4 -0.5	(-2.09 – 5.97) (9.47 – 23.9) (23.4 – 31.6) (12.8 – 31.7) (-0.44 – 5.34) (-7.62 – 6.61) (-1.10 – 6.00) (-9.70 – 8.69)	0.0002 0.24 0.32 0.37

CPs = Comparison Participants; Diff = Difference of proportions between 12-month follow-up and baseline, calculated using two sample test of proportions. ^a^ p-values for interaction between visit and case/comparison status; calculated using random effect logistic regression model by including interaction term between cases/comparison status and time point, and adjusted for age, gender and self-reported health problem in the last month to show whether non-active cases at baseline are more or less likely to become active at follow-up than comparison participant who were non-active at baseline; ^b^ Listening to radio, Reading, Watching TV; ^†^ Selling Goods.

### Performing activity without difficulty

The proportion of participants performing the different activities without any difficulty at baseline and at 12-months, and the change between the two time points are presented in Table 2. At baseline, the proportion of cases performing activities without difficulty was low for productive household activities (range 16.1–55.1%), productive outdoor activities (range 19.1–31.6%), and paid work (range 25.5–27.2%). The exception was leisure/social activities (range 46.8–73.7%), Table 2. However, there was strong evidence that trichiasis surgery improves the ability of trichiasis cases to perform all the activities investigated without difficulty. The increases for productive activities ranged from 33.1% to 65.9%, for paid work they ranged from 58.2% to 61.3% and for leisure/social activities they ranged from 22.5% to 41.3%. The largest increases were observed in productive outdoor activities: processing agricultural products increased by 65.9% (95% CI, 61.4–70.5%; p<0.0001), farming by 63.3% (95% CI, 58.4–68.1%; p<0.0001) and fetching wood by 60.7% (95% CI, 55.5–65.9%; p<0.0001), Table 2. In contrast, the comparison participants reported very little change in their ability to perform activities without difficulty. The analysis for interaction between visit and case/comparison participant status showed that for nearly all investigated activities, cases experienced a substantial reduction in difficulty one year after trichiasis surgery, relative to the comparison participants ( Table 2).

**Table 2.** Change in performing activities without difficulty between baseline and 12 month follow-up in cases and comparison participants.

Activity	Performed activity without difficulty						
	*Baseline*		*Follow-up*		*Diff*	*(95% CI)*	*P-value ^a^*
	*n/N*	*(%)*	*n/N*	*(%)*			
***Productive household activities*** Cooking and cleaning dishes Cases CPs House cleaning Cases CPs Washing clothing Cases CPs Looking after family member Cases CPs	119/740 164/168 155/708 164/167 133/382 128/129 371/673 135/136	(16.1) (97.6) (21.9) (98.2) (34.8) (99.2) (55.1) (99.3)	485/714 157/165 550/707 159/165 320/375 110/110 623/706 168/170	(67.9) (95.2) (77.8) (96.4) (85.3) (100) (88.2) (98.8)	51.8 -2.4 55.9 -1.8 50.5 0.8 33.1 -0.5	(47.5 – 56.2) (-6.47 – 1.54) (51.6 – 60.2) (-5.33 – 1.65) (44.5 – 56.5) (-0.84 – 2.29) (28.7 – 37.6) (-2.61 – 1.72)	<0.0001 <0.0001 <0.0001 ^*c*^ 0.02
***Productive outdoor activities*** Animal rearing Cases CPs Farming Cases CPs Processing agricultural products Cases CPs Fetching wood Cases CPs Fetching water Cases CPs Shopping/Marketing Cases CPs Travelling Cases CPs	204/675 159/163 89/466 118/118 96/455 158/159 95/376 148/152 222/703 165/170 145/563 147/151 93/365 114/116	(30.2) (97.5) (19.1) (100) (21.1) (99.4) (25.3) (97.4) (31.6) (97.1) (25.7) (97.3) (25.5) (98.3)	553/666 157/163 430/522 153/162 551/633 182/190 522/607 161/168 612/756 176/185 427/536 138/143 367/458 135/140	(83.0) (96.3) (82.4) (94.4) (87.0) (95.8) (86.0) (95.8) (80.9) (95.1) (79.7) (96.5) (80.1) (96.4)	52.8 -1.2 63.3 -5.6 65.9 -3.6 60.7 -1.6 49.3 -2.0 54.0 -0.8 54.6 -1.9	(48.3 – 57.3) (-4.97 – 2.51) (58.4 – 68.1) (-9.08 - -2.20) (61.4 – 70.5) (-6.69 - -0.47) (55.5 – 65.9) (-5.49 – 2.41) (44.9 – 53.8) (-5.93 – 2.08) (48.9 – 58.9) (-4.80 – 3.10) (48.9 – 60.4) (-5.73 – 0.20)	<0.0001 <0.0001 ^*c*^ <0.0001 <0.0001 <0.0001 <0.0001 0.0001
***Paid work*** Daily labouring Cases CPs Self employment ^†^ Cases CPs	12/47 4/4 40/147 24/25	(25.5) (100) 27.2 (96.0)	33/38 1/1 129/151 27/27	(86.8) (100) 85.4 (100)	61.3 0.0 58.2 0.4	(44.8 – 77.8) - (49.1 – 67.3) (-3.68 – 11.7)	0.34 ^*c*^ 0.0001 ^*c*^
***Leisure/Social activities*** Social visits Cases CPs Attending ceremonies Cases CPs Attending social meetings Cases CPs Relaxing activities ^b^ Cases CPs Daily activities Cases CPs	441/683 146/148 110/235 59/59 65/107 31/31 137/186 62/64 665/980 196/198	(64.6) (98.6) (46.8) (100) (57.9) (100) (73.7) (96.9) (67.9) (99.0)	644/703 175/181 445/505 101/103 126/131 30/30 203/211 62/63 925/980 195/198	(91.6) (96.7) (88.1) (98.1) (96.2) (100) (96.2) (98.4) (94.4) (98.5)	27.0 -1.9 41.3 -1.9 38.3 0.0 22.5 1.5 26.5 -0.5	(22.9 – 31.2) (-5.17 – 1.24) (34.3 – 48.3) (-4.61 – 0.72) (28.3 – 48.1) - (15.7 – 29.4) (-3.72 – 6.80) (23.3 – 29.8) (-2.70 – 1.69)	0.0006 <0.0001 ^*c*^ 0.0005 ^*c*^ 0.23 0.005

CPs = Comparison Participants; Diff = Difference of proportions between 12-month follow-up and baseline, calculated using two sample test of proportions. ^a^ p-values for interaction between time point and case/comparison status; calculated using random effect logistic regression model adjusted for age, gender and self-reported health problem in the last one month to show if any increase or decrease in performing an activity without difficulty from baseline to follow-up differ between cases and comparison participants; ^b^ Listening to radio, Reading, Watching TV; ^†^ Selling Goods; ^c^ p-values calculated using random effect logistic regression model to show if any increase or decrease in proportion of cases doing an activity without difficulty differ between baseline and follow-up after adjusting for potential confounders: age, gender and self-reported health problem in the last one month. This analysis is used for variables with inadequate variability in the proportion of comparison participants for interaction analysis between time point and case/comparison status.

In a combined subgroup analysis of activities, there was a significant increase in the proportion of trichiasis cases performing productive household activities (37.4%; 95% CI, 33.4–41.4%; p=0.0007), productive outdoor activities (45.6%; 95% CI 41.8– 49.5%, p<0.0001), paid work (57.5%; 95% CI, 49.5–65.5%, p<0.0001), and leisure/social activities (25.1%; 95% CI, 21.5–28.7%, p=0.0008) without any difficulty one year after trichiasis surgery.

### Performing activity without assistance

At baseline, trichiasis cases were less likely to perform activities without assistance, than the comparison participants ( Table 3). However, one year after TT surgery, there was a significant increase in the proportion of cases who could perform activities without assistance, with the increases ranging from 6.4–7.5% for paid work, 1.4–37.8% for productive activities and 0.1–3.8% for leisure/social activities. The largest increases observed were in animal rearing 37.8% (95% CI, 33.5–42.1%; p<0.0001) and farming 23.2% (95% CI, 18.8%–27.5%; p<0.0001), Table 3. The comparison participants reported very little change in their ability to perform activities without assistance. The analysis for interaction between visit and case/comparison participant status showed that for nearly all investigated activities cases experienced a substantial reduction in need for assistance one year after trichiasis surgery, relative to the comparison participants, Table 3.

**Table 3.** Change in performing activities without assistance between baseline and 12 month follow-up in cases and comparison participants.

*Activity*	*Performed activity without assistance*						
	*Baseline*		*Follow-up*		*Diff*	*(95% CI)*	*P-value ^a^*
	n/N	(%)	n/N	(%)			
***Productive household*** ***activities*** Cooking and cleaning dishes Cases CPs House cleaning Cases CPs Washing clothing Cases CPs Looking after family member Cases CPs	642/740 167/168 629/708 166/167 344/382 129/129 564/673 135/136	(86.8) (99.4) (88.8) (99.4) (90.0) (100) (83.8) (99.3)	680/714 165/165 695/707 165/165 363/375 110/110 686/706 170/170	(95.2) (100) (98.3) (100) (96.8) (100) (97.2) (100)	8.4 0.6 9.5 0.6 6.8 0.0 13.4 0.7	(5.58 – 11.4) (-0.57 – 1.76) (6.95 – 12.0) (-0.57 – 1.76) (3.26 – 10.2) - (10.3 – 16.4) (-0.70 – 2.17)	<0.0001 <0.0001 0.001 <0.0001
***Productive outdoor*** ***activities*** Animal rearing Cases CPs Farming Cases CPs Processing agricultural products Cases CPs Fetching wood Cases CPs Fetching water Cases CPs Shopping/Marketing Cases CPs Travelling Cases CPs	366/675 144/163 341/466 116/118 401/455 159/159 345/376 150/152 599/703 169/170 546/563 150/151 342/365 115/116	(54.2) (88.3) (73.2) (98.3) (88.1) (100) (91.8) (98.7) (85.2) (99.4) (96.9) (99.3) (93.7) (99.1)	613/666 162/163 503/522 162/162 626/633 190/190 602/607 168/168 734/756 185/185 527/536 143/143 448/457 140/140	(92.0) (99.4) (96.4) (100) (98.9) (100) (99.2) (100) (97.1) (100) (98.3) (100) (98.0) (100)	37.8 11.1 23.2 1.7 10.8 0 7.4 1.3 11.9 0.6 1.4 0.7 4.3 0.9	(33.5 – 42.1) (5.97 – 16.1) (18.8 – 27.5) (-0.63 – 4.02) (7.68 – 13.8) - (4.55 – 10.3) (-0.50 – 3.13) (9.00 – 14.8) (-0.56 – 1.74) (-0.44 – 3.12) (-0.63 – 1.96) (1.53 – 7.13) (-0.82 – 2.54)	<0.0001 <0.0001 <0.0001 <0.0001 <0.0001 0.19 0.02
***Paid work*** Daily labouring Cases CPs Self employment ^†^ Cases CPs	44/47 4/4 132/147 25/25	(93.6) (100.0) (89.8) (100)	39/39 1/1 147/151 27/27	(100) (100) (97.3) (100)	6.4 0.0 7.5 0.0	(-0.61 – 13.4) - (2.03 – 13.1) -	- 0.03
***Leisure/Social activities*** Social visits Cases CPs Attending ceremonies Cases CPs Attending social meetings Cases CPs Relaxing activities ^b^ Cases CPs Daily activities Cases CPs	664/683 148/148 226/235 59/59 106/107 31/31 178/186 63/64 955/980 198/198	(97.2) (100) (96.2) (100) (99.1) (100) (95.7) (98.4) (97.4) (100)	697/703 181/181 497/505 103/103 130/131 30/30 209/210 62/63 969/980 198/198	(99.1) (100) (98.4) (100) (99.2) (100) (99.5) (98.4) (98.9) (100)	1.9 0.0 2.25 0.0 0.1 0.0 3.8 0.0 1.5 0.0	(0.52 – 3.34) - (-0.44 – 4.93) - (-2.18 – 2.53) - (0.76 – 6.89) - (0.24 – 2.62) -	0.02 0.33 0.91 0.04 0.02

CPs = Comparison Participants; Diff = Difference of proportions between 12 month follow-up and baseline, calculated using two sample test of proportions. ^a^ p-values calculated using random effect logistic regression model to show if any increase or decrease in proportion of cases doing an activity without assistance differ between baseline and follow-up after adjusting for potential confounders: age, gender and self-reported health problem in the last one month. This analysis is chosen as the change in assistance (between baseline and follow-up) data in the comparison participants have inadequate variability for interaction analysis between time point and case/comparison status; ^b^ Listening to radio, Reading, Watching TV; ^†^ Selling Goods. Dashed lines indicate that the proportion variability in the data is inadequate for such analysis.

In a combined subgroup analysis of activities, there was an increase one year after trichiasis surgery in the proportion of trichiasis cases performing productive household activities (5.7%; 95% CI, 3.7–7.6%, p<0.0001), productive outdoor activities (5.8%; 95% CI, 3.9–7.6%, p<0.0001), paid work (6.6%; 95% CI 2.2–11.0%, p=0.03), and leisure/social activities (1.9%; 95% CI, 0.6–3.1%, p=0.02) without assistance.

### Effect of vision change on difficulty and assistance

We examined whether the increase in proportion of case performing an activity without difficulty or assistance showed a trend across the three groups of vision changes, Table 4. The improvement in processing agricultural products (p=0.006), cooking and cleaning dishes (p=0.03), animal rearing (p=0.03) and making social visits (p=0.03) without difficulty was significantly greater among people who experienced improvement in their visual acuity, compared to those with unchanged or worse vision. For most other activities, the proportion of cases performing the activity without difficulty and their ability to perform an activity without assistance, improved by similar degrees for all three vision change groups (p-value for trend >0.10; Table 4). 

**Table 4.** Change in performing activities without difficulty and assistance in cases by vision change at 12 months.

*Activity*	Performed activity without difficulty							Performed activity without assistance						
	Vision better		Vision same		Vision worse		*P-value ^a^*	Vision better		Vision same		Vision worse		*P-value ^a^*
	*Diff*	*95% CI*	*Diff*	*95% CI*	*Diff*	*95% CI*		*Diff*	*95% CI*	*Diff*	*95% CI*	*Diff*	*95% CI*	
***Productive household activities*** Cooking and cleaning dishes House cleaning Washing clothing Looking after family member	55.2 60.1 55.1 40.3	(48.1 – 62.3) (53.5 – 67.7) (44.3 – 65.9) (32.4 – 48.3)	53.1 54.0 44.9 29.0	(46.8 – 29.4) (47.9 – 60.6) (36.3 – 53.6) (22.9 – 35.1)	41.8 51.0 58.5 31.3	(31.2 – 52.4) (40.9 – 61.4) (46.1 – 70.9) (20.8 – 41.7)	0.03 0.09 0.70 0.11	9.7 10.9 8.0 14.8	(4.31 – 15.1) (6.41 – 15.4) (1.95 – 14.0) (9.08 – 20.5)	6.9 7.9 5.3 11.0	(3.20- 10.6) (4.44 – 11.2) (0.48 – 10.2) (7.06 – 14.8)	9.5 10.9 9.8 16.7	(2.19 – 16.8) (4.70 – 17.2) (1.56 – 18.1) (9.11 – 24.3)	0.91 0.70 0.85 0.95
***Productive outdoor activities*** Animal rearing Farming Processing agricultural products Fetching wood Fetching water Shopping/Marketing Travelling	59.3 57.0 74.7 63.8 53.9 55.2 53.4	51.7 – 66.9) (48.0 – 66.1) (68.8 – 81.6) (55.1 – 72.4) (46.4 – 61.4) (46.3 – 64.0) (43.7 – 63.1)	51.5 67.4 63.3 63.4 48.4 53.5 56.6	(45.1 – 57.9) (60.9 – 74.1) (56.4 – 70.2) (56.1 – 70.8) (42.0 – 54.8) (46.5 – 60.5) (48.1 – 65.0)	45.2 63.6 57.4 49.8 43.7 53.2 50.4	(34.2 – 55.9) (52.6 – 74.6) (46.4 – 68.3) (37.0 – 62.7) (33.4 – 54.0) (42.1 – 64.3) (36.8 – 64.1)	0.03 - 0.006 0.12 0.05 0.78 0.56	38.3 24.2 16.2 7.8 15.3 3.6 5.3	(30.4 – 46.2) (16.1 – 32.3) (9.89 – 22.5) (2.69 – 12.9) (9.63 – 21.0) (-0.90 – 8.06) (0.15 – 10.4)	39.3 24.8 7.0 5.4 7.6 0.3 3.1	(33.4 – 45.2) (18.8 – 30.8) (3.24 – 10.7) (1.76 – 9.03) (4.27 – 11.0) (-0.92 – 1.60) (0.43 – 5.82)	33.7 17.8 10.6 12.2 15.6 0.2 3.5	(23.8 – 43.7) (8.06 – 27.6) (3.67 – 14.4) (40.5 – 20.3 (8.35 – 22.8) (-4.07 – 4.52 (-4.99 – 12.1)	0.90 0.83 0.42 0.74 0.85 0.33 0.27
***Paid work*** Daily labouring Self employment ^†^	80.6 55.0	(54.7 – 1.06) (36.1 – 73.9)	59.3 59.8	(39.1 – 79.4) (47.8 – 71.7)	52.4 55.8	(12.1 – 92.6) (33.7 – 77.8)	0.27 0.88	16.7 6.8	(-4.42 – 37.7) (-6.03 – 19.6)	3.6 6.5	(-3.30 – 10.4) (0.25 – 12.7)	0 10.4	- (-4.48 – 25.4)	- 0.73
***Leisure/Social activities*** Social visits Attending ceremonies Attending social meetings Relaxing activities ^e^ Daily activities	32.9 43.2 40.4 23.6 27.5	(25.6 – 40.2) (30.5 – 55.9) (20.2 – 60.5) (11.5 – 35.6) (21.6 – 33.2)	27.4 39.3 31.8 20.9 24.9	(21.8 – 33.1) (29.2 – 49.4) (18.9 – 44.7) (11.3 – 30.4) (20.3 – 29.6)	18.0 41.6 49.7 23.5 28.4	(8.44 – 27.5) (28.8 – 26.4) (28.6 – 70.9) (7.58 – 39.4) (21.0 – 35.8)	0.03 0.96 0.91 0.42 0.71	3.4 1.7 3.6 7.2 2.4	(0.61 – 6.23) (-2.52 – 5.91) (-3.30 – 10.4) (-1.23 – 15.6) (0.08 – 4.75)	0.33 3.0 -1.5 0 1.3	(-1.1 – 1.75) (1.32 – 7.32) (-4.46 – 1.43) - (-0.03 – 2.71)	3.0 1.3 0 5.0 0	(-0.41 – 6.34) (-4.21 – 6.85) - (-1.75 – 11.7) -	0.77 0.75 - - 0.20

Diff = Difference of proportions between 12 month follow-up and baseline, calculated using two sample test of proportions. ^a^ p-values for interaction between time point and vision change at 12-month follow-up in cases alone; calculated using random effect logistic regression model adjusted for age, gender and self-reported health problem in the last one month to show if any increase or decrease in performing an activity without difficulty/assistance among the cases show a trend across the three group of vision changes: better, same and worse. P values particularly indicate if there is a trend of a larger difference with worse vision; ^e^ Listening to radio, Reading, Watching TV; ^†^ Selling Goods. Dashed lines indicate that the proportion variability in the data is inadequate for such analysis.

### Effect of recurrence on participation, difficulty and assistance

At 12-month follow-up, 131/980 (13.4%) cases had TT recurrence. We examined if there is a difference in activity participation between cases with and without recurrence at 12-month, Table 5. This showed no significant difference in almost all activities. The exception is the daily labouring activity, where significantly less proportion of cases with recurrence at 12-month participated than those without recurrence (-6.1%; p=0.023 Table 5). We examined whether the increase in proportion of cases performing an activity without difficulty or assistance differed among those with and without TT recurrence at 12-month ( Table 5). For most activities, the proportion of cases performing the activity without difficulty improved by similar degree regardless of the presence of TT recurrence; except the improvement in shopping/marketing (p=0.006), and engaging in self-employment activities (p=0.007) without difficulty was significantly greater among those without recurrence, compared to those with recurrence. Similarly, the improvement in farming (p=0.019) without assistance was significantly greater among cases with no recurrence at 12-months. Otherwise, for most other activities, the proportion of cases performing the activity without assistance, improved by similar degrees regardless of TT recurrence ( Table 5).

**Table 5.** Change in performing activities, and performing activities without difficulty and assistance in cases by recurrence status at 12 months.

*Activity*	Performed activity in the last week					Performed activity without difficulty					Performed activity without assistance				
	No Recurrence		Recurrence		P-value *^a^*	No Recurrence		Recurrence		P-value *^a^*	No Recurrence		Recurrence		*P-value ^a^*
	*Diff*	*95% CI*	*Diff*	*95% CI*		*Diff*	*95% CI*	*Diff*	*95% CI*		*Diff*	*95% CI*	*Diff*	*95% CI*	
***Productive household activities*** Cooking and cleaning dishes House cleaning Washing clothing Looking after family member	-2.9 -.04 -1.3 4.0	(-7.1 – 1.2) (-4.7 – 4.0) (-5.9 – 3.3) (-0.3 – 8.3)	-0.8 1.2 1.5 -0.8	(-11.0 – 10.0) (-8.4 – 10.7) (-10.1 – 13.1) (-12.6 – 11.1)	0.35 - 0.60 0.29	53.1 57.1 51.7 32.8	(48.5 – 57.8) (52.6 – 61.7) (45.4 – 58.0) (28.1 – 37.6)	43.3 47.7 42.1 35.3	(31.0 – 55.7) (35.0 – 60.3) (24.2 – 60.0) (22.2 – 48.4)	0.20 0.11 0.093 0.95	8.1 9.2 7.3 14.1	(5.0 – 11.1) (6.6 – 11.8) (3.5 – 11.2) (10.9 – 17.4)	11.4 11.2 2.3 7.5	(2.0 – 20.8) (2.7 – 19.8) (-4.9 – 9.4) (-1.3 – 16.3)	0.98 0.28 0.49 0.068
***Productive outdoor activities*** Animal rearing Farming Processing agricultural products Fetching wood Fetching water Shopping/Marketing Travelling	-0.7 7.5 18.7 24.0 5.8 -3.9 10.4	(-5.0 – 3.7) (2.8 – 12.3) (14.1 – 23.4) (19.4 – 28.6) (1.7 – 9.9) (-8.6 – 0.8) (5.7 – 15.0)	-2.3 -6.1 13.7 21 3.0 3.0 3.8	(-14.2 – 9.6) (-18.2 – 5.9) (1.8 – 25.7) (8.8 – 32.4) (-7.9 – 14.0) (-9.0 – 15.1) (-8.1 – 15.8)	0.69 - 0.38 0.57 0.44 0.22 0.29	52.7 63.5 66.8 60.7 49.2 56.1 54.9	(47.9 – 57.4) (58.4 – 68.7) (62.0 – 71.7) (55.1 – 66.3 (44.4 – 53.9) (51.0 – 61.3) (49.0 – 61.1)	53.6 60.0 59.5 60.5 50.5 35.7 51.8	(40.4 – 66.9) (45.8 – 74.3) (46.0 – 73.0) (46.9 – 74.1) (38.0 – 62.8) (19.2 – 52.2) (36.1 – 67.6)	0.82 0.51 0.27 0.65 0.93 0.006 0.64	38.2 24.2 10.7 7.5 11.7 1.9 4.3	(33.6 – 42.7) (19.8 – 29.0) (7.5 – 14.0) (4.5 – 10.5) (8.6 – 14.7) (0.07 – 3.7) (1.4 – 7.3)	35.0 14.3 10.8 6.8 13.3 -3.1 4.1	(22.1 – 48.0) (1.7 – 26.9) (1.5 – 20.0) (-1.6 – 15.0) (4.9 – 21.7) (-9.3 – 3.1) (-4.4 – 12.6)	0.37 0.019 0.18 0.17 0.86 0.10 0.57
***Paid work*** Daily labouring Self employment ^†^	-0.1 0.8	(-2.0 – 1.8) (-2.6 – 4.3)	-6.1 -2.3	(-11.1 – -1.1) (-9.9 – 5.3)	0.023 0.32	56.4 62.4	(37.8 – 74.9) (53.2 – 71.7)	90.0 18.8	(71.0 – 100.0) (-18.1 – 53.7)	- 0.007	5.4 7.8	(-1.9 – 12.7) (1.8 – 13.8)	10.0 6.2	(-8.6 – 28.6) (-5.6 – 18.1)	- -
***Leisure/Social activities*** Social visits Attending ceremonies Attending social meetings Relaxing activities ^e^ Daily activities	2.6 26.7 2.6 2.7 -	(-1.8 – 6.9) (22.3 – 31.2) (-0.5 – 5.7) (-1.1 – 6.6) -	-2.3 32.1 1.5 0.8 -	(-12.8 – 8.3) (21.0 – 43.0) (-6.2 – 9.2) (-8.5 – 10.0) -	0.42 0.32 0.79 0.66 -	28.1 41.9 38.4 21.4 26.1	(23.6 – 32.5) (34.5 – 49.0) (27.9 – 49.0) (14.2 – 28.6) (22.6 – 29.6)	20.9 36.8 36.6 30.6 29.0	(9.9 – 31.9) (15.9 – 57.6) (8.1 – 65.1) (9.6 – 51.7) (20.0 – 38.0)	0.18 0.64 0.54 0.70 0.71	2.2 2.0 1.1 3.1 1.4	(0.7 – 3.8) (-0.9 – 4.9) (-1.0 – 3.2) (0.07 – 6.2) (0.2 – 2.7)	-0.06 3.8 -6.2 8.7 1.5	(-4.0 – 3.9) (-3.5 – 11.2) (-18.1 – 5.6) (-2.8 – 20.2) (-2.1 – 5.1)	0.16 - - - 0.85

Diff = Difference of proportions between 12-month follow-up and baseline, calculated using two sample test of proportions. ^a^ p-values for interaction between time point and recurrence at 12-month follow-up in cases alone; calculated using random effect logistic regression model adjusted for age, gender and self-reported health problem in the last one month to show if any increase or decrease in participating in an activity, and performing an activity without difficulty/assistance among the cases is different among those with and without TT recurrence at 12-month; ^**e**^ Listening to radio, Reading, Watching TV; ^†^ Selling Goods. Dashed lines indicate that the proportion variability in the data is inadequate for such analysis; except for daily activities participation where data is not available.

### Ocular pain, trichiasis surgery and its impact on daily living

At baseline 968/980 (98.8%) of the trichiasis cases seen again at the 12-month follow-up had ocular pain. Of these, 61% felt it often or constantly. In a multivariable analysis, baseline major trichiasis (OR, 1.54; 95% CI, 1.21–1.95; p=0.0004), female gender (OR, 1.63; 95% CI, 1.22–2.19; p=0.0011), and reports of other health problem in the last month (OR, 2.27; 95% CI, 1.77–2.92; p<0.0001), were significantly associated with increased frequency of ocular pain in trichiasis cases. In addition, cases with major TT at baseline were more likely to report ocular pain interfering with participation in productive household (OR, 1.65; 95% CI, 1.30–2.10; p<0.0001), paid or agricultural (OR, 1.48; 95% CI, 1.16–1.88; p=0.0015), and social activities (OR, 1.29; 95% CI, 1.01–1.64; p=0.043), than cases with minor TT. 

One year after trichiasis surgery, the proportion of cases experiencing ocular pain reduced from 98.9% to 33.7% (Proportion difference, 65.2%; 95% CI, 62.1–68.1%; p<0.0001). In contrast, the proportion of comparison participants with ocular pain increased by 7.6% (95% CI, 3.4%–11.7%; p<0.0001) at follow-up. At baseline a considerable number of trichiasis cases reported that ocular pain interfered with personal care (31.2%), sleep (70.0%), participation in productive household activities (79.2%), paid or agricultural work (83.6%), and social activities (53.6%), Table 6. However, one year after trichiasis surgery the proportion experiencing interfering pain dropped substantially, Table 6. There were very significant decreasing trends in the frequency of ocular pain and its interference with sleep, engagement in personal care, productive household, paid or agricultural and social activities among TT cases, Table 6. In multivariable analysis, recurrent TT during the 12-month period was strongly associated with increased likelihood of ocular pain at 12-months (OR, 1.83; 95% CI, 1.30–2.56; p=0.0005); and increasing frequency of ocular pain was strongly associated with reduced participation in productive household activities (OR, 1.84; 95% CI, 1.15–2.97; p=0.012) in TT cases.

**Table 6.** Change in impact of ocular pain on daily living between baseline and 12 months after trachomatous trichiasis surgery in cases and comparison participants.

*Activity*	Cases							Comparison participants							*P value* ^b^
	*Baseline* *N=1000*		*12 Months* *N=980*		*Diff* ^†^	*95% CI*	*P value* ^a^	*Baseline* *N= 200*		*12 Months* *N=198*		*Diff* ^†^	*95% CI*	*P value* ^a^	
	*n*	*(%)*	*n*	*(%)*				*n*	*(%)*	*n*	*(%)*				
***Ocular pain*** No Occasionally Often Constantly	12 369 343 256	(1.2) (37.7) (35.0) (26.1)	650 261 54 15	(66.3) (26.6) (5.5) (1.5)	-65.2	(-68.1 – -62.1)	<0.0001	196 2 0 0	(99.0) (1.0) (0.0) (0.0)	181 15 2 0	(91.4) (7.6) (1.0) (0.0)	7.6	(3.43 – 11.7)	0.0004	<0.0001
***Ocular pain interfered personal care*** No Occasionally Often Constantly	688 207 70 35	(68.8) (20.7) (7.0) (3.5)	962 10 7 1	(98.2) (1.0) (0.71) (0.1)	-28.6	(-31.6 – -25.6)	<0.0001	198 2 0	(99.0) (1.0) (0.0) (0.0)	198 0 0 0	(100) (0.0) (0.0) (0.0)	-0.1	(-2.4 – 0.38)	-	-
***Ocular pain disturbed sleep*** No Occasionally Often Constantly	300 332 301 67	(30.0) (33.2) (30.1) (6.7)	909 57 14 0	(92.8) (5.8) (1.4) (0.0)	-62.9	(-66.0 – -59.4)	<0.0001	198 1 0 1	(99.0) (0.5) (0.0) (0.5)	196 2 0 0	(99.0) (1.0) (0.0) (0.0)	0.0	-	-	0.0001
***Ocular pain interfered productive household*** ***activities*** No Occasionally Often Constantly	208 426 290 76	(20.8) (42.6) (29.0) (7.6)	862 90 23 5	(88.0) (9.2) (2.3) (0.5)	-67.2	(-70.2 – -63.7)	<0.0001	198 0 0 2	(99.0) (0.0) (0.0) (1.0)	197 1 0 0	(99.5) (0.5) (0.0) (0.0)	-0.5	(-2.21 – 1.20)	-	0.02
***Ocular pain interfered paid or agricultural work*** No Occasionally Often Constantly	164 502 248 86	(16.4) (50.2) (24.8) (8.6)	891 66 18 5	(90.9) (6.7) (1.8) (0.5)	-74.6	(-77.2 – -71.3)	<0.0001	198 0 1 1	(99.0) (0.0) (0.5) (0.5)	198 0 0 0	(100) (0.0) (0.0) (0.0)	-1.0	(-2.4 – 0.38)	-	-
***Ocular pain interfered social activities*** No Occasionally Often Constantly	464 385 94 57	(46.4) (38.5) (9.4) (5.7)	944 25 7 4	(96.3) (2.6) (0.7) (0.4)	-49.9	(-52.7 – -46.0)	<0.0001	198 0 1 1	(99.0) (0.0) (0.5) (0.5)	197 1 0 0	(99.5) (0.5) (0.0) (0.0)	-0.5	(-2.21 – 1.20)	-	0.02

^†^ Diff = proportion difference from two sample test of proportions of those with any level of problem secondary to ocular pain between baseline and 12 month follow-up; creating binary variable after combining those with “occasionally”, “often” and “constantly” responses.

^a^ p-values calculated using random effect ordinal logistic regression model adjusted for age, gender and self-reported health problem in the last one month to show if there is a trend in decrease in proportion of cases and comparison participants (separately) with negative impact of ocular pain on daily living between baseline and follow-up; ^b^ p-values for interaction between time point and case/comparison status; calculated using random effect ordinal logistic regression model by including interaction term between cases/comparison status and visit; and adjusted for age, gender and self-reported health problem in the last one month to show if any increase or decrease in proportion of cases and comparison participants with no pain or no negative impact of ocular pain on daily living between baseline and follow-up significantly differ between cases and comparison participants. Dashed lines indicate that the proportion variability in the data is inadequate for such analysis.

## Discussion

The relationship between trachoma and poverty is likely to be bidirectional, with poverty being both a cause and consequence of trachoma ^4^. Trichiasis can cause pain and visual impairment, which may limit participation in productive activity and execution of tasks, resulting in disability. In settings similar to this study, engagement in non-paid household, outdoor and agricultural activities make a substantial economic contribution to household wealth. In fact, participation in non-paid or non-monetized household and outdoor activities is estimated to make a $16 trillion “invisible” monetary contribution to global economic output and between 20% and 60% of national GDP in some regions ^9, 18^. Women, who are three times more affected by TT than men, undertake most of the unpaid household and care work ^8^. Here we explored whether TT surgery improves participation in and performance of activities.

Overall, there was little evidence of a major change in the proportion of people participating in a wide range of daily task and social activities. This might be anticipated for a number of reasons. Firstly, most of these activities are necessary for and intrinsic to life in the communities from which the participants live. People with trichiasis still need to do most of these activities, despite their disability. This implies that measuring participation alone would not capture the negative effects of trachoma and other NTDs on functioning and daily living, and tools that can capture the level of difficulty and amount of assistance required in executing an activity should be used. Secondly, within a household a particular activity might be the responsibility of specific family members. Therefore, the TT case that had not been doing that particular activity at baseline might not necessarily take over that task from someone else after surgery. 

The key changes we found were in how able cases were to perform the activities they were already engaged in. Performing productive activities without difficulty or assistance increases independence and productivity, which will potentially have a considerable social return and reduce financial strains on the household, through increased contributions. We found strong evidence that trichiasis surgery, regardless of change in vision, could have a major effect on improving functioning and performance in productive and leisure/social activities.

Firstly, trichiasis surgery significantly improved the ability of trichiasis cases to perform productive activities without difficulty. At baseline, the proportion of cases performing paid work, productive household and outdoor agricultural activities without any difficulty ranged between 16% and 51%. These increased to between 33% and 66% one year after trichiasis surgery. In contrast, comparison participants reported little difficulty with tasks, and this proportion was largely unchanged. The largest improvements were seen in the ability to execute agricultural activities, such as processing agricultural products (66%) and farming (63%). Increased capacity to perform agricultural activities can increase agricultural productivity, which is a major driver for improved food security and human development in sub-Saharan Africa ^19^. Our study findings were consistent with findings of a study conducted in Southern Ethiopia which measured the physical functioning of trichiasis patients before and six months after surgery ^7^. In this earlier study, at baseline 61.1% of trichiasis cases reported difficulty in physical functioning including performing day-to-day farming activities. However, six months after trichiasis surgery, the percentage of participants reporting difficulty in physical functioning reduced to 32.6%.

Moreover, executing an activity without difficulty is critical, as it has a positive effect on both physical and mental wellbeing, thereby improving quality of life ^9^. A qualitative study in Niger found that women affected by TT were not able to help or care for their families and have diminished spirit and social status ^20^. However, TT surgery substantially improved their quality of life and social reintegration ^20^. In our study, the proportion of trichiasis cases attending ceremonies, social meetings and relaxing activities without any difficulty increased by more than 22%, indicating the holistic positive effect trichiasis surgery has on the social integration and the day to day lives of people affected by trichiasis. This in turn would have a broader positive impact in building self-esteem or a sense of dignity through engagement in society and income generating activities.

Although the greatest increase in some of the activities was reported by those with improved vision, the improvement in executing most of the productive tasks without difficulty was apparent even among trichiasis cases who had not experienced an improvement in vision, suggesting that treating the pain caused by trichiasis through surgery can improve the ability to perform tasks. Similarly, the improvement in executing most of the productive tasks without difficulty was seen even among cases who had recurrent TT. This is probably related to the fact that most of the recurrent trichiasis is caused by few lashes (more than 90% had minor recurrent TT) that are probably less likely to impede the ability to execute an activity ^13^. On the other hand, recurrent TT was associated with persistent ocular pain, which in turn was associated with less participation in productive activities following surgery. Higher frequency of reported pain and increasing interference in productive and social activities was significantly associated with baseline TT severity, indicating that trichiasis severity greatly determines the functional capabilities of TT cases. These data together suggest that prompt and high quality TT surgery provides considerable economic and social benefits to patients with severe TT. 

Secondly, we found that trichiasis surgery improved the ability of operated individuals to perform productive activities without assistance. One year after trichiasis surgery, the proportion of trichiasis cases performing productive activities without assistance significantly improved by about 6% to 17% from the baseline. The proportion of TT cases who could execute productive activities such as animal rearing and farming without any kind of assistance increased by 38% and 23%, respectively. This increment was found even without improved visual acuity and regardless of the presence of recurrent TT. The reduced need for assistance would be expected to lead to increased productivity of the individuals, as well as less time spent by other household members supporting them, leading to time being released to engage with other activities. Both elements are anticipated to contribute to a reduction in household poverty.

Thirdly, there is evidence that reducing ocular pain through trichiasis surgery improves engagement in productive paid and agricultural activities. At baseline we found that trichiasis cases suffered from ocular pain, which was reported to interfere with their personal care, sleep and social participation. It also affected perceived involvement in productive household, paid or agricultural activities, suggesting that trichiasis pain contributes to disability and impedes productivity. One year after trichiasis surgery, the frequency of ocular pain interfering in productive activities markedly reduced, and the proportion of trichiasis cases that reported ocular pain interfering with productive activities significantly reduced by more than two thirds. Similar results have been reported in a longitudinal study, in which the proportion of trichiasis cases with pain and discomfort reduced by more than 90% six months after trichiasis surgery ^7^. Chronic pain may lead to depression and other mental disorders. Alleviating the ocular pain through surgery might also have a positive effect in improving mental health. Further studies are needed to explore the effect of trachomatous trichiasis and its surgical management on mental health of affected individuals.

This study is the first large comparative longitudinal study to measure the impact of trichiasis surgery on participation, difficulty and assistance needs of people living with trichiasis on a wide range of productive and leisure/social activities. The same interviewers collected data at both baseline and follow-up to ensure questionnaires were administered in a standard way. The study has a number of limitations. The interviewers were not masked to the trichiasis status of the participants. We cannot exclude the possibility of response bias. In an earlier report, more than 90% of the cases in this study reported satisfaction with the outcomes of TT surgery ^13^. In low-income settings such as this study’s area, participation in productive activities would be affected by seasonality of activities. The baseline and 1-year follow-up data were collected during the same time of the year: the dry season when communities are less engaged in productive activities and more engaged in leisure/social activities compared to other times of the year. This might explain the lack of difference in the proportion of both cases and comparison participants engaged in most of the productive activities between baseline and follow-up. Activity participation, difficulty and assistance were measured through self-report. Time spent in different activities was not measured, as pilot studies demonstrated difficulty estimating and recalling this. The pain impact questionnaire was not a standardized tool, but was developed to be locally relevant through community focus group discussion. Type I error is possible from multiple comparisons.

## Conclusions

There was strong evidence that TT surgery improves the ability of TT cases in executing productive and leisure/social activities. TT surgery substantially increased the proportion of patients who perform productive and leisure/social activities without difficulty and assistance. Trichiasis surgery effectively treated ocular pain and discomfort, which in turn improved engagement in productive paid and agricultural activities. These data together suggest that trichiasis surgery could have a major effect in improving productivity and contributing to household income and wealth. An unprecedented effort is under way to scale-up trichiasis surgical programmes. Providing prompt surgical intervention to prevent visual loss will also improve overall socio-economic engagement of affected individuals and contribute to the wealth of affected communities.

## Data availability

The data referenced by this article are under copyright with the following copyright statement: Copyright: © 2017 Habtamu E et al.

This study is a collaboration between the Amhara Regional Health Bureau, The Carter Center Ethiopia, and LSHTM. The Amhara Regional Health Bureau requires that all data sharing requests are reviewed and approved by them. Data is available to any researcher under reasonable request. To facilitate the data access process, please contact: ethics@lshtm.ac.uk, who will provide guidance for accessing the data from the Amhara Regional Health Bureau.
