# Supplementary material for: Impact of trichiasis surgery on daily living: A longitudinal study in Ethiopia
Source: Wellcome Open Res. 2017 Dec 6;2:69. Originally published 2017 Aug 22. [Version 2] doi: 10.12688/wellcomeopenres.11891.2 (PMC5686479; doi:10.12688/wellcomeopenres.11891.2)
Supplement: Peer review history

# Peer review: Referee response for version 2

**DOI:** 10.21956/wellcomeopenres.14402.r28745

**Published:** 2017-12-07

**Authors:** Charlotte Hanlon

**Affiliations:** 1 Department of Psychiatry, School of Medicine, College of Health Sciences, Addis Ababa University, Addis Ababa University, Ethiopia; 2 Health Service and Population Research Department,Centre for Global Mental Health, Institute of Psychiatry, Psychology and Neuroscience, Kings College London, London, UK

The authors have fully accommodated my comments.

I have read this submission. I believe that I have an appropriate level of expertise to confirm that it is of an acceptable scientific standard.

# Peer review: Referee response for version 1

**DOI:** 10.21956/wellcomeopenres.12849.r26704

**Published:** 2017-11-13

**Authors:** Charlotte Hanlon

**Affiliations:** 1 Department of Psychiatry, School of Medicine, College of Health Sciences, Addis Ababa University, Addis Ababa University, Ethiopia; 2 Health Service and Population Research Department,Centre for Global Mental Health, Institute of Psychiatry, Psychology and Neuroscience, Kings College London, London, UK

This is well-conducted study which addresses an important research question, examining the impact of trichiasis surgery on daily living in a rural subsistence farming setting in Ethiopia. The methods are clearly presented and the measures appear to have captured relevant aspects of rural life. The analyses are appropriate. The authors are to be congratulated for conducting such a rigorous study. I only have minor comments for the authors to consider.

  

1. The term ‘leisure’ does not seem fully appropriate for this setting, where most of the ‘leisure’ activities are social obligations which people need to fulfill in order to maintain bonds of reciprocity. The domain is extremely important, but perhaps ‘social activities’ would be a more accurate descriptor.
2. It is very interesting that participation in activities did not distinguish cases and the comparison participants, but only level of difficulty in engaging in those activities. This underlines the importance of using measures that capture level of difficulty as well as participation, a point which might be worth emphasizing.
3. One of the most interesting findings is that improvement in visual acuity was not associated with improved capacity to carry out activities without difficulty or needing assistance. I was not clear why you used a different type of analysis to look at the effect of pain (and not the same as the analysis used for recurrence of trichiasis). I also thought this could be an area where you might want to recommend further study, for example, to explore how depression might mediate some of the disability associated with pain (and may not be immediately reversible when pain is alleviated + may cause additional suffering and disability) – comorbid depression is common in most chronic disorders, especially when associated with pain, and has been shown to contribute to a greater proportion of disability than the physical health condition.
4. There were a lot of statistical comparisons and so the chance of type 1 error might need to be mentioned, although the consistent pattern of associations was convincing.
5. There is a typo – page 16, column 2, para 2, half-way down – ‘impend’ should be ‘impede’.

 

I have read this submission. I believe that I have an appropriate level of expertise to confirm that it is of an acceptable scientific standard.

# Response

**Published:** 2017-11-22

**Authors:** Esmael Habtamu Ali

**Affiliations:** London School of Hygiene & Tropical Medicine, UK

Many thanks for your thoughtful review. You have raised very important points in your comments. Please kindly see below the point by point response we have given to each of your comments. 

1) We agree with your comment that the leisure activities category consists of mostly social activities. However, “Leisure activities” is the term used in the ‘Stylised Activity List’ tool developed for the World Bank Living Standard Measurement Survey (the tool we used to collect the data) to categorise those activities.

We have amended the “Leisure activities” term into “Leisure/Social activities” wherever this occurred throughout the manuscript to address this comment. 

2) Thank you for pointing this out. We completely agree. We have added the following sentence on page 16, first column, paragraph 2, line 7.

*“This implies that measuring participation alone would not capture the negative effects of trachoma and other NTDs on functioning and daily living, and tools that can capture the level of difficulty and amount of assistance required in executing an activity should be used”.*

3) The aim of the pain data analysis was to show the effect of trichiasis surgery on alleviating pain, and the effect of this on daily living. Our data showed that TT surgery significantly reduces pain, and even where there was pain after trichiasis surgery, the frequency of that pain was reduced significantly after trichiasis surgery, which in turn has a positive effect on sleep and participation in productive and social activities. This is the reason why we do not want to analyse the pain data as binary (was it present? YES/NO) as we did for the recurrence data, but rather as ordered categorical data.

If we clearly understood your point on depression and pain, our data suggest that surgery alleviates ocular pain markedly, which might also suggest pain associated depression might be relieved. However, we agree that further studies are needed to elicit this association, and have discussed what you have mentioned in your comment. We have included the following sentences on page 17, first column, at the end of the first paragraph.  

*“Chronic pain may lead to depression and other mental disorders. Alleviating the ocular pain through surgery might also have a positive effect in improving mental health. Further studies are needed to explore the effect of trachomatous trichiasis and its surgical management on mental health of affected individuals”.*

4) We have included the following sentence on page 17, first column, at the end of paragraph 2.

*“Type I error is possible from multiple comparisons”.*

5) Thank you for picking this up. This is now corrected to “impede”.

# Peer review: Referee response for version 1

**DOI:** 10.21956/wellcomeopenres.12849.r25861

**Published:** 2017-10-09

**Authors:** Muhammad Mansur Rabiu

**Affiliations:** 1 Consultant, Noor Dubai Foundation, Dubai, United Arab Emirates

The study is a well-designed and conducted research that adds to the increasing evidence of the benefit of lid survey for trachomatous trichiasis beyond the possible improvement of vision and prevention of visual deterioration.

 However, one aspect of the article that highlights some limitation of the study is the fact that the Interviewers that collected the data were not masked to the cases and control groups. Probably, a possible approach may be to have had the interviews for both the cases and comparison groups in the health facilities with interviewers conducting the interview behind a screen so that they cannot see the faces of the cases and control ( as cases of trachomatous trichiasis and even post-surgical cases can be recognized from their faces).

I have read this submission. I believe that I have an appropriate level of expertise to confirm that it is of an acceptable scientific standard.

# Response

**Published:** 2017-10-27

**Authors:** Esmael Habtamu Ali

**Affiliations:** London School of Hygiene & Tropical Medicine, UK

Many thanks for your thoughtful review. You have highlighted an important potential limitation of this study. We agree with this and had indicated this to be a limitation of the study in the last paragraph of the discussion: *“The interviewers were not masked to the trichiasis status of the participants. We cannot exclude the possibility of response bias”.*

We agree with the suggestion that interviewing both the cases and comparison groups in the health facilities with interviewers conducting the interview behind a screen would have reduced this risk of bias. However, this would not have been a practical option and we think would not have been culturally acceptable in this context. The comparison groups were recruited and followed at their homes while the cases were recruited at a health facility for the following two reason.

Firstly, this study was part of a larger, previously reported study where the household economic welfare of trichiasis cases and health controls (comparison participants) was compared and studied. Three methods were used to measure household economic welfare: (a) Collecting data on a broad set of asset-based wealth indicators and then relative household economic poverty was determined by principal component analysis (PCA, (b) Self-rated wealth, and (c) Peer-rated wealth.  Most of the asset based wealth indicator data needs to be collected through observation which requires the data collectors to go to the houses of the cases and comparison participants at baseline and interacting with them which again might create a chance for the data collectors to recognise some of the participants from their answers even in an interview done behind a screen. In addition, the peer rated wealth data was collected through interviewing three peers of both the cases and the comparison participants, which again required visiting the villages of all participants.

Secondly, bringing the comparison groups to a health facility both at baseline and follow-up would have been logistically difficult. Trichiasis cases were mainly motivated to come to the health facility to receive surgical management for their trichiasis and for subsequent surgical follow-ups, while the comparison groups would not have such motivation.

Thank you again for reviewing our manuscript.
